# Validation of the orthostatic hypotension knowledge, attitudes, and practices questionnaire and investigation of influencing factors: a cross-sectional study

**DOI:** 10.3389/fpubh.2025.1561758

**Published:** 2025-10-14

**Authors:** Qin Zou, Rao Li, Qian Chen, Bo Gu

**Affiliations:** ^1^Department of Dermatology and Venerology, West China Hospital, Sichuan University, Chengdu, China; ^2^West China School of Nursing, Sichuan University, Chengdu, China; ^3^Department of Endocrinology and Metabolism, West China Hospital, Sichuan University, Chengdu, China; ^4^Center of Gerontology and Geriatrics, West China Hospital, Sichuan University, Chengdu, China; ^5^Nursing Key Laboratory of Sichuan, West China Hospital, Sichuan University, Chengdu, China; ^6^Nephrology and Urology Ward, West China Hospital, Sichuan University, Chengdu, China

**Keywords:** orthostatic hypotension, knowledge, attitudes, practices, questionnaire, validation

## Abstract

**Aims:**

This study aimed to develop and validate tools to assess inpatients’ knowledge, attitudes, and practices concerning orthostatic hypotension (OH) and to explore the influencing factors.

**Design:**

This study was a cross-sectional validation study conducted from July to December 2024.

**Methods:**

This research employed a cross-sectional study design, in which the OH knowledge, attitudes, and practices questionnaire (KAPQ) was administered to hospitalized patients. The Delphi expert consultation, Spearman correlation analysis and factor analysis were used to evaluate and validate the questionnaire. The current status and influencing factors of patients’ KAPQ scores were subsequently analyzed.

**Results:**

Of the 1,488 patients who completed the validated questionnaire, 12.77% experienced OH in the hospital. The KAPQ had a Cronbach’s alpha of 0.97, with exploratory factor analysis revealing three factors explaining 74% of its variance. The item-total correlations for the KAPQ ranged from 0.55 to 0.82. Test–retest reliability was evaluated with a Spearman rank correlation coefficient of 0.89. Individuals with a college education or higher had a lower risk of low KAPQ scores than did those with a high school education or less (OR: 0.67, 95% CI: 0.55–0.82). Receiving health education on OH also reduced the risk of low scores (OR: 0.36, 95% CI: 0.30–0.45). Additionally, a higher FES-I score was linked to a decreased risk of low KAPQ scores (OR: 0.98, 95% CI: 0.97–0.99).

**Conclusion:**

The self-reported KAPQ scale demonstrated robust reliability and validity coefficients, effectively assessing knowledge, attitudes, and practices regarding OH among inpatients. Inpatients who had received health education related to OH, had higher FES-I scores, possessed higher educational levels, and achieved comparatively elevated KAPQ scores concerning OH.

## Introduction

Orthostatic hypotension (OH) is a common cardiovascular disease, that is defined as a postural drop in systolic blood pressure of ≥20 mmHg or a drop in diastolic blood pressure of ≥10 mmHg (1, 2). The prevalence of OH in community-dwelling older people and cardiac patients is over 20% (3–5), and OH affects as many as 30–68% of institutionalized patients (6). OH can cause numerous symptoms of insufficient blood perfusion, such as dizziness, headache, fatigue, blurred vision and other issues, thus affecting the life of patients (2, 7). It is a common disorder in the older adults, debilitated patients, and patients with neurological diseases, and is associated with markedly increased risk of fall, stroke, or even death (8). Researchers have reported that OH is associated with hypotension-related hospitalizations or emergency department visits, and leads to diabetes and a poor prognosis for future cardiovascular events (9, 10). However, outside of hospital settings, the serious consequences of OH in patients are usually ignored (4).

Preventing the occurrence of OH and reducing its complications can relieve its impact on patients and improve their quality of life. Health education interventions for medical staff and self-management of patients, including lifestyle changes, are the main nondrug treatment measures used to prevent OH (8, 11). Some studies have shown that health education about disease-related knowledge may improve patients’ understanding of corresponding diseases, to reduce risk behavior in daily life (12–14). The knowledge structure and behavior of patients are directly related to effective OH management.

In the epistemological framework, individuals acquire knowledge and skills through learning processes, subsequently forming pertinent beliefs and attitudes, which in turn influence and promote individual behavior. Accordingly, the deficiency in patients’ specific knowledge frameworks will have a direct effect on the practices and outcomes within related disciplines (15). Previous studies have shown that better patients knowledge is, associated with improved self-management ability (16). Xu et al. (12) reported that knowledge, attitudes, and practice intervention measures for older adults can effectively manage hypertension. For patients with OH, a deficiency in their understanding of OH-related knowledge can directly influence their behavior. Therefore, comprehending the knowledge, attitudes, and practices of patients with OH is highly important for clinicians in devising effective intervention strategies for managing this condition.

Our literature review reveals that current research tools predominantly concentrate on assessing the knowledge, attitudes, and practices of diverse populations impacted by hypertension, heart failure, and other medical conditions, alongside evaluating nurses’ knowledge, attitudes, and practices regarding orthostatic hypotension (17, 18). Nevertheless, prior studies have insufficiently investigated the knowledge, attitudes, and practices related to orthostatic hypotension among hospitalized patients. At present, there is an absence of an assessment instrument specifically designed to evaluate the knowledge, attitudes, and practices of hospitalized patients concerning orthostatic hypotension. The aim of this study was to develop and validate assessment tools for gauging the OH knowledge, attitudes, and practices of inpatients, and for examining the factors influencing them, with the objective of informing patients about future preventive interventions.

## Methods

### Patient population and sample size

Inclusion and exclusion criteria: Hospitalized patients of both sexes aged more than 18 years and who were willing to participate in the study were included. Participants who could write or communicate independently in Chinese were also included. Individuals who experienced severe mental illness, rendering them incapable of responding or engaging in communication, were excluded. In the literature reviewed by Daniel J. et al., determining the absolutely necessary minimum sample size is often deemed impractical. Within the context of factor analysis, a sample size of 100 is considered poor, 200 is deemed average, 300 is regarded as good, 500 is very good, and 1,000 or more is considered excellent (19). Given the specific circumstances of this project team, convenience sampling was employed, resulting in a sufficient number of cases. This study included 1,524 inpatients from 9th ward at West China Hospital, Sichuan University, who were selected through convenience sampling. Ultimately, 1,488 eligible cases were included in the analysis.

### Outcome measures

#### KAPQ

The OH knowledge, attitudes, and practices questionnaire (KAPQ) was developed through a comprehensive literature review (1, 8, 20, 21), followed by a Delphi expert consultation and subsequent discussions with patients. The selection of questions was further refined based on statistical analysis and a preliminary survey. The item reduction for the KAPQ of OH is as follows:

(1) Development of item banks: Through a comprehensive review of the literature, consultations with clinical nursing experts, and interviews with patients experiencing OH, an initial pool of 33 items was constructed, encompassing three dimensions: knowledge, attitudes, and practices (Supplementary document 1).
(2) Delphi Expert Consultation: A panel of 24 experts, all possessing intermediate or higher professional titles and expertise in OH, were assembled to participate in the Delphi expert consultation process. The panel of 24 experts encompassed a diverse range of fields, including clinical medicine, clinical nursing, rehabilitation, management, psychology, and health education (Supplementary document 1). Experts were solicited to assess the dimensions of the proposed scale, as well as the precision and significance of the item descriptions. Experts completed two rounds of the Delphi expert consultation. The participants had a mean age of 44.38 ± 7.91 years and an average of 22.13 ± 10.73 years of professional experience. The questionnaire achieved an effective response rate of 96%, with an average opinion submission rate of 30.5% across the two rounds. The expert authority coefficient for the consultation was 0.85. The evaluation employed a 5-point Likert scale, where 1 indicates “not important,” 2 indicates “not very important,” 3 indicates “moderately important,” 4 indicates “somewhat important,” and 5 indicates “very important.” The scale’s content was refined based on the experts’ feedback, and the authority coefficient was subsequently calculated. Following two rounds of expert consultation, as well as the subsequent modification and screening of the questionnaire items, the statements “taking hypoglycemic drugs can cause postural hypotension” and “taking antianxiety drugs can cause postural hypotension” were removed. Consequently, the finalized version of the KAPQ on OH was established, comprising 29 items that encompass knowledge, attitudes, and practices (Supplementary document 2).
(3) In the preliminary survey, a convenience sampling method was employed to select 31 inpatients from a ward at West China Hospital of Sichuan University for a pre-investigation; this was undertaken to reassess and enhance the accuracy and feasibility of the written descriptions, thereby aligning the questionnaire more closely with clinical practice. The critical ratio method, the correlation coefficient method between items and the total score, and factor analysis were utilized to eliminate unsuitable items.

The final questionnaire comprises a total of 29 questions distributed across three domains: knowledge, attitudes, and practices. Specifically, it includes 13 items pertaining to the knowledge dimension, 7 items addressing the attitudes dimension, and 9 items related to the practices dimension (Supplementary document 2). The questionnaire is a self-rating scale with a total score ranging from 29 to 145 points. The total score is determined by aggregating the scores of all the items. A higher total score indicates a higher level of knowledge, attitudes and practices. Each item is evaluated via a 5-point Likert scale, ranging from 1 to 5 points, ranging from strongly disagree, disagree, somewhat agree, agree and strongly agree. Patients took 5 to 8 min to complete the questionnaire.

#### Barthel Index (BI)

In this study, the activities of daily living (ADL) of patients were evaluated via the BI scale, a globally recognized assessment tool (22–24). The Barthel Index (BI) scale encompasses 10 specific tasks and assigns scores based on the time required or the level of assistance needed by the patient. The cumulative score ranges from 0 to 100, with lower scores indicating a higher degree of nursing dependency.

#### Falls efficacy scale international (FES-I)

The 16-item FES-I is extensively utilized to evaluate patients’ concerns regarding falls (25, 26). This scale was endorsed in the 2022 world guidelines for falls and is structured into two sections: indoor and outdoor activities (27). It comprises a total of 16 items, with 10 items pertaining to indoor activities and 6 items addressing outdoor activities. The scale employs a 4-level scoring system, assigning 0 to 3 points for each item. Consequently, the total score ranges from 16 to 64 points. A higher score indicates a greater degree of patient fear, which correlates with a reduced efficiency in fall prevention.

#### Data collection

**Figure 1:**
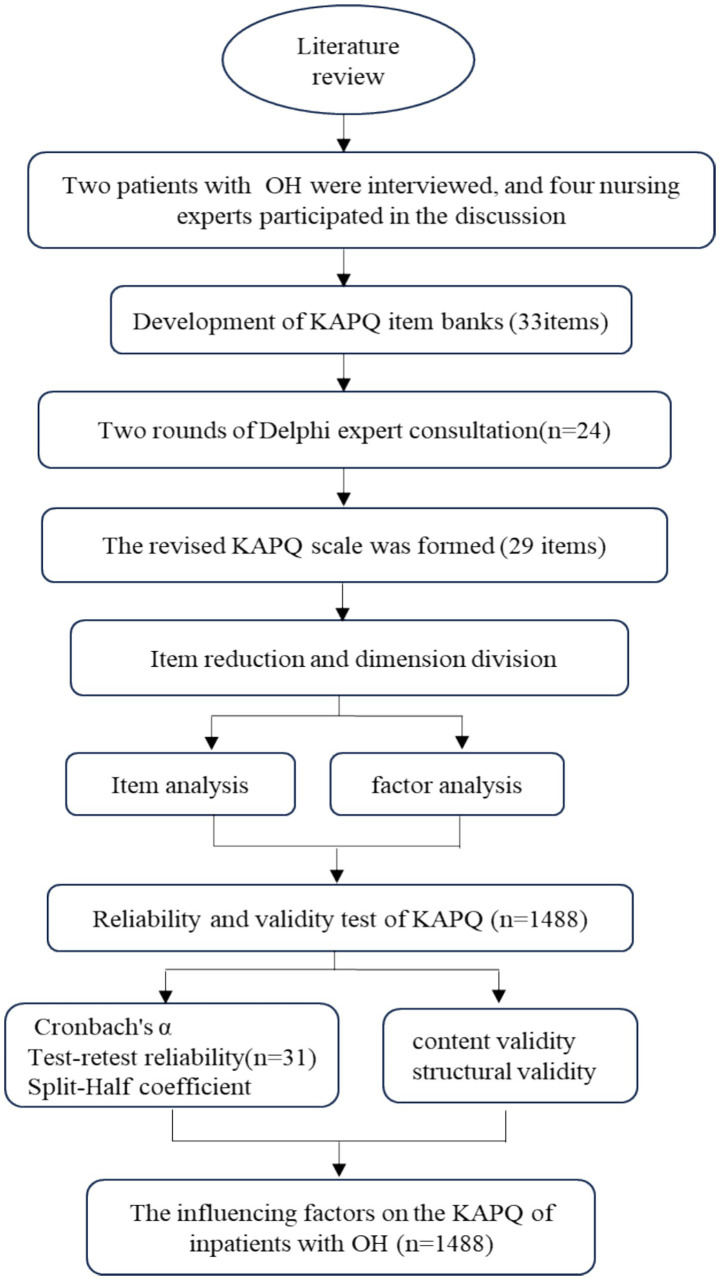
The research process of this study.

Patients information, including age, sex, marital status, education, employment status, primary economic source, history of falls within the past year, presence of hypertension, body mass index (BMI) and blood pressure, was collected when patients were admitted to the hospital. Blood pressure was measured after 5 min in the supine position and within 3 min of assuming the standing position (8). Patients were invited to complete the KAPQ, BI, and EFS-I 16 on the first day of their admission. The research process of this study is shown in Figure 1.

### Data analysis

The data were analyzed via SPSS software version 26.0 (SPSS Inc., Chicago, Illinois) and R Project for Statistical Computing (version 4.5.1). Demographic variables were characterized by summarizing the frequency and percentage for categorical variables, and the mean, standard deviation (SD), and median for continuous variables.

The critical ratio, correlation analysis, factor analysis and Cronbach’s alpha coefficient were used to identify the items on the KAPQ questionnaire. The reliability of the KAPQ questionnaire was evaluated by internal consistency, split–half reliability and test–retest reliability. Cronbach’s alpha was used to assess internal consistency. Split-half reliability was assessed via Guttman’s half coefficient and the Spearman-Brown coefficient. Test–retest reliability was evaluated by the Spearman rank correlation coefficient for scores obtained at baseline and 2 weeks later. Validity was tested as content validity and construct validity the content validity of the KAPQ was evaluated by experts. The construct validity was assessed by exploratory factor analysis (EFA) and confirmatory factor analysis (CFA). The Kaiser–Meyer–Olkin (KMO) test and Bartlett test were used to assess the feasibility of performing the factor analysis. Construct validity was also evaluated through Spearman’s rank correlation analysis between the total KAPQ scores and individual item scores.

Ordered logistic regression analysis was employed to examine the factors influencing inpatients with OH. Given that the scores from the knowledge and practices scale of the study population did not follow a normal distribution, they were categorized into four equal-length intervals based on the interquartile range. These intervals included Q_25_ (≤99 points), Q_25-50_ (>99 points and ≤114 points), Q_50-75_ (>114 points and ≤125 points), and Q_75-100_ (>125 points). Using the Q_25_ interval score as a reference, an ordered logistic regression analysis was conducted to examine the factors involved. The analysis adjusted for covariates such as age, sex, body mass index (BMI), literacy level, alcohol consumption, history of falls, the BI score, the FES-I, the Charlson comorbidity index, the presence of hypertension, heart disease, diabetes, chronic kidney disease, solid tumors, cerebrovascular disease, postural hypotension, polypharmacy, and health education related to OH. A bilateral test level was employed, and a *p*-value of less than 0.05 was deemed statistically significant.

## Results

### Basic information of the research subjects

A total of 1,524 patients were enrolled in this study. A total of 1,488 patients successfully completed the validated questionnaire, yielding a response rate of 97.64%. The incidence of OH among hospitalized patients was determined to be 12.77%. The demographic characteristics of the study population are detailed in Table 1.

**Table 1:** Characteristics of the participants (*N* = 1,488).

Items	*n*(%)	Items	Mean ± SD, Range, n(%)
Sex		Age(year)	64.11 ± 17.15(18,98)
Male	878(59.00)	<65	620(41.67)
Female	610(41.00)	≥65	868(58.33)
Marital status		Body mass index	23.46 ± 3.47(13.67,38.13)
Single	249(16.73)	FES-I score	15.22 ± 12.25(0,48)
Married	1,239(83.27)	BI score	67.01 ± 34.80(0,100)
Education background		KAPQ score	110.93 ± 20.36(30,144)
High school and below	962(64.65)	Knowledge score	47.54 ± 10.96(13,65)
College degree and higher	526(35.35)	Attitudes score	27.82 ± 5.30(7,35)
Fall within a year		Practices score	35.57 ± 6.68(9,45)
Yes	184(12.37)	KAPQ score	
No	1,304(87.63)	Q_25_ ≤ 99	385(25.87)
Hypertension		99<Q_25-50_ ≤ 114	383(25.74)
Yes	885(59.48)	114<Q_50-75_ ≤ 125	355(23.86)
No	603(40.52)	125<Q_75-100_	365(24.53)
Orthostatic hypotension		Health education related to orthostatic hypotension	
Yes	190(12.77)	Yes	498(33.47)
No	1,298(87.23)	No	990(66.53)

FESI-16, Falls Efficacy Scale International; KAPQ, Knowledge, Attitudes, and Practices Questionnaire; BI, Barthel Index.

### Reliability

In this study, the Cronbach’s alpha coefficient for the total KAPQ score was determined to be 0.97, indicating a high level of internal consistency. The Cronbach’s alpha coefficients for the individual dimensions of knowledge, attitudes, and practices were found to be 0.97, 0.95, and 0.93, respectively. Test–retest reliability was evaluated via the Spearman rank correlation coefficient, which yielded a value of 0.89. The correlation coefficients for individual items between the two measurements ranged from 0.65 to 0.99. The Spearman-brown coefficient and Guttman split-half coefficient of the KAPQ total score were both 0.80 and 0.79, respectively (Table 2).

**Table 2:** Reliability of the KAPQ (*N* = 1,488).

Spearman rank correlation coefficient (*n* = 31)				Dimension	Cronbach’s α	Spearman-Brown coefficient	Guttman split-half coefficient
Item1	0.88^**^	Item16	0.97^**^	Knowledge dimension score	0.97	0.93	0.92
Item2	0.81^**^	Item17	0.89^**^	Attitudes dimension score	0.95	0.89	0.86
Item3	0.84^**^	Item18	0.87^**^	Practices dimension score	0.93	0.91	0.89
Item4	0.76^**^	Item19	0.96^**^	Total score	0.97	0.80	0.79
Item5	0.80^**^	Item20	0.89^**^	—	—	—	—
Item6	0.96^**^	Item21	0.92^**^	—	—	—	—
Item7	0.77^**^	Item22	0.94^**^	—	—	—	—
Item8	0.89^**^	Item23	0.94^**^	—	—	—	—
Item9	0.86^**^	Item24	0.89^**^	—	—	—	—
Item10	0.81^**^	Item25	0.91^**^	—	—	—	—
Item11	0.86^**^	Item26	0.89^**^	—	—	—	—
Item12	0.84^**^	Item27	0.65^**^	—	—	—	—
Item13	0.88^**^	Item28	0.89^**^	—	—	—	—
Item14	0.89^**^	Item29	0.99^**^	—	—	—	—
Item15	0.85^**^	Total score	0.89^**^	—	—	—	—

** = correlation is significant at the 0.01 level (2-tailed).

### Validity

Following two rounds of Delphi expert consultation, it was concluded that the questionnaire possesses strong content validity. The results of the KMO test and Bartlett’s test of sphericity showed that factor analysis was suitable for the KAPQ. Three factors were identified, accounting for 74% of the variance in the KAPQ. Varimax rotation was used for the factor loading analysis, and the factor loading of each item on the Three factors was > 0.4. The findings from the CFA indicated that the dimensions of knowledge, attitudes, and practices, along with each individual item, demonstrated robust discriminant validity (Supplementary document 3). The results revealed that the item-total correlation coefficient of the KAPQ ranged from 0.55 to 0.82. Additionally, the Spearman correlation coefficients between individual item scores and dimension scores ranged from 0.73 to 0.92, further supporting the instrument’s structural validity (Table 3).

**Table 3:** Spearman rank correlation coefficients of the item–total correlations of the KAPQ (*N* = 1,488).

Items	Total score	Knowledge	Items	Total score	Attitudes	Items	Total score	Practices
Item1	0.70^**^	0.80^**^	Item14	0.76^**^	0.84^**^	Item21	0.76^**^	0.84^**^
Item2	0.72^**^	0.83^**^	Item15	0.78^**^	0.87^**^	Item22	0.73^**^	0.82^**^
Item3	0.72^**^	0.81^**^	Item16	0.80^**^	0.90^**^	Item23	0.73^**^	0.85^**^
Item4	0.76^**^	0.83^**^	Item17	0.77^**^	0.89^**^	Item25	0.59^**^	0.73^**^
Item5	0.78^**^	0.81^**^	Item18	0.76^**^	0.89^**^	Item25	0.55^**^	0.73^**^
Item6	0.82^**^	0.86^**^	Item19	0.75^**^	0.88^**^	Item26	0.58^**^	0.81^**^
Item7	0.82^**^	0.87^**^	Item20	0.66^**^	0.79^**^	Item27	0.63^**^	0.81^**^
Item8	0.82^**^	0.86^**^	—	—	—	Item28	0.66^**^	0.87^**^
Item9	0.81^**^	0.86^**^	—	—	—	Item29	0.68^**^	0.85^**^
Item10	0.82^**^	0.86^**^	—	—	—	—	—	—
Item11	0.81^**^	0.87^**^	—	—	—	—	—	—
Item12	0.81^**^	0.86^**^	—	—	—	—	—	—
Item13	0.80^**^	0.92^**^	—	—	—	—	—	—

** = correlation is significant at the 0.01 level (2-tailed). *p* < 0.001 for all.

### Influencing factors on the KAPQ score of inpatients with OH

**Figure 2:**
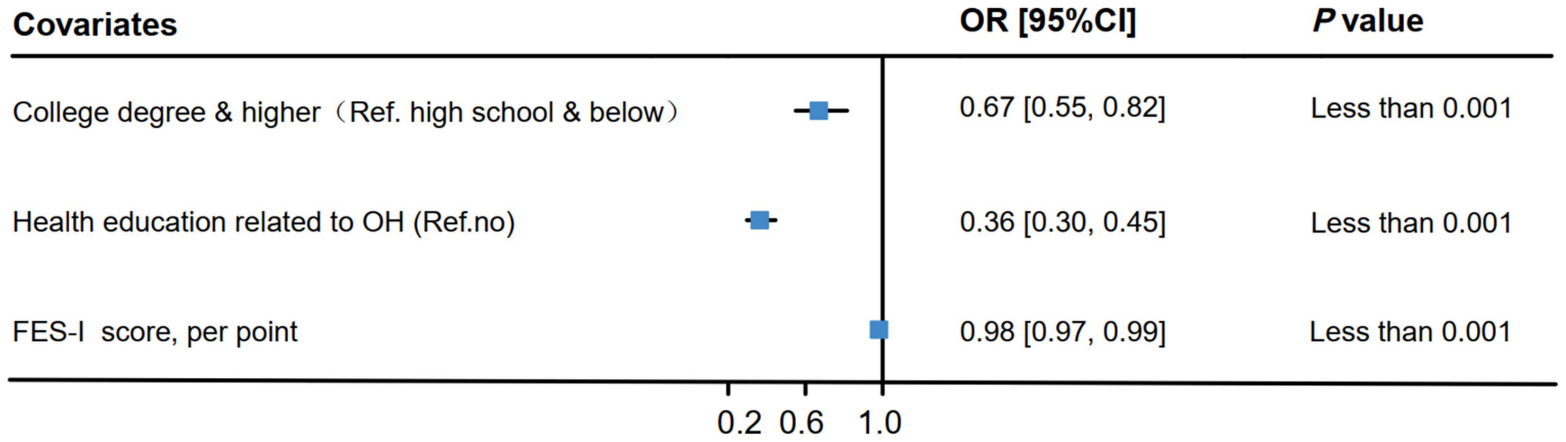
Analysis of factors influencing the KAPQ score.

The developed ordered logistic regression model met the proportional odds assumption (χ^2^ = 44.89, *p* = 0.27), and no multicollinearity was detected among the independent variables included in the analysis (tolerance > 0.1, variance inflation factor < 10). Consequently, ordered logistic regression was employed in this study for factor analysis. The results indicated that individuals with a college education or higher had a lower risk of low knowledge, attitudes and practices scores than did those with a high school education or less (OR: 0.67, 95% CI: 0.55–0.82). Similarly, those who received health education on OH had a lower risk of low scores in the Q_25_ range than those who did not (OR: 0.36, 95% CI: 0.30–0.45). Additionally, a higher FES-I score was associated with a reduced risk of low knowledge and practices scores in the Q_25_ range (OR: 0.98, 95% CI: 0.97–0.99) (Figure 2).

## Discussion

This study examined OH incidence in inpatients, developed and tested a questionnaire on their knowledge, attitudes and practices (KAPQ) regarding OH; and analyzed the influencing factors of the KAPQ. OH typically manifests following an abrupt transition to an upright posture. However, it may also occur postprandially, during physical exertion, or after prolonged periods of standing (1). The condition primarily results from cerebral hypoperfusion, with key symptoms including generalized weakness, dizziness or vertigo, blurred vision, and, in severe instances, loss of consciousness or syncope (28). In this study, utilizing the established criteria for measuring OH (29, 30), the prevalence among hospitalized patients was determined to be 12.77%, which aligns with findings from related research (21, 28). According to an investigation conducted by Yang (31) the prevalence of postural hypotension among hospitalized patients with cardiovascular diseases in a specific region of China is approximately 23.8%, which exceeds the prevalence reported in this study. This discrepancy may be attributed to differences in the surveyed populations. Yang’s research focused on inpatients diagnosed with cardiovascular diseases (31). In contrast, this study predominantly examines individuals with diabetes, dermatological conditions, and a range of other health issues, resulting in a reduced incidence of OH among the inpatients assessed.

In this study, the scores of inpatients with varying levels of knowledge and practices were roughly equivalent, averaging approximately 25%. Approximately 50% of patients had an intermediate KAPQ score between 99 and 125. Consistent with our findings, a survey conducted by Haron et al. (32) in Malaysia reported that 57.4% of the Malay older adults exhibited moderate knowledge and attitudes regarding salt intake. In this study, approximately 24.5% of patients showed positive attitudes toward OH prevention, which was much higher than the 3% reported by Tahani et al. (33) for knowledge and attitudes related to other geriatric diseases. This discrepancy may be attributed to variations in survey populations and content.

Based on literature reports on postural hypotension management, the content of the KAPQ scale used in this study was compiled (1). There are several methods for selecting items. To determine the retention of items, the appropriate method should be selected according to the research purpose, followed by the reliability and validity of the scale. In this study, the development of the questionnaire items was informed by two rounds of Delphi expert consultations, followed by a presurvey and statistical analyses, ultimately identifying three dimensions comprising 29 items. The primary focus of the questionnaire encompassed symptoms, risk factors, hazards, preventive measures, and the acquisition of knowledge related to OH (1, 2, 4). The knowledge dimension items are systematically organized based on the associated symptoms and prevalent causes of OH (1, 2). This includes knowledge pertaining to the symptoms of OH (Items 10–13), the causes (Items 4–8), and medication-related information (Items 1–3). The attitudes dimension primarily encompasses patients’ assessments of the risks posed by OH, such as the potential for falls, the significance of management implementation, their apprehension regarding OH, and their attitudes towards learning and acquiring knowledge related to OH (Items 14–21) (4). The behavioral dimension primarily encompasses the commitment to implement appropriate interventions to prevent OH (Items 22–27), the resolve to gain the necessary knowledge for its prevention (Item 28), and the apprehension regarding the potential of medication to induce OH (Item 29) (20).

The findings of this study demonstrated that the KAPQ scale exhibits robust reliability and validity, aligning with the scientific principles, rationality, and applicability inherent to self-rating scales. The majority of literature has focused predominantly on the knowledge, beliefs, and practices of patients with hypertension, while insufficient attention has been given to content related to OH. Furthermore, reports concerning assessment tools specifically designed to evaluate the knowledge, attitudes, and practices of patients with OH are lacking (17, 34, 35). Current reports are limited to domestic studies on OH knowledge and practices among health care professionals in China (36). In this study, the Cronbach’s alpha coefficient for the KAPQ was determined to be 0.97, with the coefficients for the individual domains of knowledge, attitudes, and practices being 0.97, 0.95, and 0.93, respectively, thereby demonstrating high internal consistency. A cohort of thirty-one patients completed the questionnaire at two separate intervals, 2 weeks apart, and the correlation between the two sets of results indicated satisfactory test–retest reliability for the scale. Furthermore, the Spearman-brown coefficient and the Guttman split-half coefficient were calculated to be 0.80 and 0.79, respectively. The findings consistently demonstrated that the scale possesses strong reliability and serves as an effective and dependable instrument. Expert evaluation and patient completion of the questionnaire substantiated its robust content validity. The correlation coefficients between individual items and the overall score ranged from 0.73 to 0.87, providing additional evidence for the instrument’s structural validity. Furthermore, the questionnaire requires only 5 to 8 min to complete. The questionnaire items are clear, comprehensible, and reliable. It serves as a valuable tool for staff to assess the knowledge, attitudes, and practices associated with mitigating OH in hospital settings. Furthermore, it facilitates the development of targeted intervention strategies to prevent OH in inpatients.

In this study, the analysis of the ordered logistic regression model revealed that inpatients who had received health education on OH, those with elevated FES-I scores, and individuals with higher educational attainment presented significantly higher scores in knowledge, attitudes, and practices related to OH. This enhanced understanding was positively associated with the adoption of preventive measures against the risk of OH. OH is a prevalent yet frequently overlooked condition, attributed to a variety of etiological factors and is associated with asthenic symptoms, diabetes, cardiovascular disease, and cognitive impairment (4). Although diabetes has been identified as a risk factor for OH (30), this study revealed no significant increase in KAPQ scores among diabetic patients. Furthermore, the risk factors for OH did not appear to be directly correlated with the KAPQ scores of individuals diagnosed with diabetes. This lack of correlation may be attributed to the fact that the majority of diabetic patients in this study received disease-related health education during hospitalization and consequently exhibited greater attention to disease management. OH can result in symptoms such as unexplained dizziness, dyspnea, fatigue, and blurred vision. OH can significantly impact patients’ daily activities, delay disease recovery, and increase the risk of adverse events, including falls, mortality, stroke, heart failure, and atrial fibrillation (8, 21, 37). Consequently, the timely identification and management of OH in hospitalized patients are crucial. Health education is an effective strategy to increase patients’ quality of life and prevent both short and long-term complications. In this study, patients who presented elevated fall efficacy scores also presented increased KAPQ scores concerning OH, which aligns with findings reported in previous studies (28). In general, a greater understanding of OH among patients facilitates more effective management of associated behaviors, thereby promoting better control of the symptoms and reducing the incidence of comorbidities. Shrestha et al. (38) conducted a study revealing a correlation between the educational level of hypertensive patients and their knowledge, beliefs, and practices regarding the condition. The findings indicated that patients with higher educational attainment demonstrated greater knowledge and belief scores. Similarly, Li et al. (16) examined a hypertensive population in a rural region of Southeast China, and reported that knowledge, attitudes and practices associated with hypertension were linked to the rates of awareness, treatment, and control of the disease. Following health education on OH, the patient’s knowledge structure is likely to improve, thereby facilitating the adoption of preventive strategies, such as lifestyle modifications, to effectively mitigate the risk of OH.

Limitations: Criterion validity was not subjected to a comparative analysis owing to the lack of analogous instruments for evaluating the knowledge, attitudes, and practices of inpatients with OH. The participants used to assess the reliability and validity of the KAPQ questionnaire was the same as that employed for logistic regression analysis in this study due to the substantial sample size, which presented challenges for division. This investigation was conducted as a cross-sectional study and did not assess the impact of interventions on patients with OH. Consequently, the research team intends to implement preventive interventions for patients with OH and subsequently evaluate their effectiveness.

## Conclusion

In this study, the self-reported KAPQ scale demonstrated robust reliability and validity coefficients, effectively assessing the knowledge, attitudes, and practices regarding OH among inpatients. Inpatients who had received health education related to OH, had higher FES-I scores, and higher educational levels achieved relatively elevated KAPQ scores concerning OH. This correlation indicates that health education concerning OH among inpatients may be effective in preventing the incidence of OH.

## Data Availability

The original contributions presented in the study are included in the article/Supplementary material, further inquiries can be directed to the corresponding authors.
