# Supplementary material for: Validation of the orthostatic hypotension knowledge, attitudes, and practices questionnaire and investigation of influencing factors: a cross-sectional study
Source: Front Public Health. 2025 Oct 14;13:1561758. doi: 10.3389/fpubh.2025.1561758 (PMC12560239; doi:10.3389/fpubh.2025.1561758)
Supplement: Supplementary file 1 [file Table_1.DOCX]

**Supplementary document 1: Orthostatic hypotension knowledge, attitudes, and practices questionnaire initial items pool and Delphi expert consultation results**

**1. An initial item pool for the Orthostatic Hypotension Knowledge, Attitudes, and Practices Questionnaire was developed, comprising a total of 33 items (Table 1).**

| Table 1：Orthostatic Hypotension KAPQ Initial Items Pool | |
| --- | --- |
| number | **Knowledge dimension** |
| 1 | Taking antidepressants and anti-anxiety drugs can cause OH. |
| 2 | Taking sedative drugs can cause OH. |
| 3 | Taking hypnotic drugs can cause OH. |
| 4 | Taking hypoglycemic drugs can cause OH. |
| 5 | Insufficient water intake can cause OH. |
| 6 | Taking diuretics can cause OH. |
| 7 | Sudden positional changes (sitting up or getting up) can lead to OH. |
| 8 | OH can lead to falls. |
| 9 | Certain chronic conditions such as cardiovascular disease or diabetes increase the risk of OH. |
| 10 | Post-bath or heavy sweating activities increase the risk of OH. |
| 11 | Weakness and fatigue increase the risk of OH. |
| 12 | OH may occur when you feel dizzy after changing positions or standing for a long time. |
| 13 | OH may occur when you experience dizziness, blurred vision, or a black haze upon changing positions or standing for a long time. |
| 14 | OH may occur when you experience weakness of the lower extremities after changing positions or standing for a long time. |
| 15 | OH may occur when you experience nausea or vomiting after changing positions or standing for a long time. |
| 16 | OH may occur when you experience dyspnea after changing positions or standing for a long time. |
|  | **Attitudes Dimension** |
| 17 | I believe that OH can cause falls. |
| 18 | I believe that implementing management measures can lower the risk of OH. |
| 19 | I believe that OH management is important. |
| 20 | I believe that OH does not pose a significant risk to health. |
| 21 | I'd like to learn about the risk factors of OH. |
| 22 | I'd like to learn about the symptoms of OH. |
| 23 | I'd like to learn about the preventive measures for OH. |
| 24 | I'm worried about getting OH. |
|  | **Practices Dimension** |
| 25 | I will proactively take measures to prevent OH and lower risk of falls. |
| 26 | I will stand up slowly to give my body an adjustment to prevent OH. |
| 27 | I will drink plenty of water, especially after excessive sweating to prevent OH. |
| 28 | I will wear lower limb compression socks to prevent OH. |
| 29 | I will avoid drinking alcohol. |
| 30 | I will do daily exercise. |
| 31 | I will follow my health care provider's advice to prevent OH. |
| 32 | I will learn how to prevent OH. |
| 33 | If I take medication, I will concern about medication affecting my blood pressure and causing dizziness. |

**2. Delphi expert consultation results**

The Delphi expert consultation invited a total of 25 experts from various fields, encompassing clinical medicine, clinical nursing, rehabilitation, management, and health education. These experts were geographically distributed across eastern, central, and western China, and included two advisors for graduate students. Their professional experience spanned from 3 to 35 years, with a mean of 22.13±10.73 years, while their ages ranged from 29 to 55 years, with a mean age of 44.38±7.91 years. In the initial round of consultation, all 25 experts participated, whereas 24 experts completed the second round (Table 2).

Table 2：Expert Basic Information(n=24)

| Groups | | n/ Mean±standard deviation | Percentage (%) |
| --- | --- | --- | --- |
| Age |  | 44.38±7.91 | — |
| Education | Bachelor's degree | 13 | 54.17 |
|  | Master's degree | 7 | 29.17 |
|  | Doctorate degree | 4 | 16.67 |
| Professional field | Clinical medicine | 5 | 20.83 |
|  | clinical nursing | 11 | 45.83 |
|  | rehabilitation | 2 | 8.33 |
|  | management | 4 | 16.67 |
|  | health education | 2 | 8.33 |
| academic title | Lecturer | 6 | 25 |
|  | Associate professor | 16 | 66.67 |
|  | Professor | 2 | 8.33 |
| professional experience |  | 22.13±10.73 | — |

The questionnaire achieved an effective response rate of 96%, with an average opinion submission rate of 30.5% across the two rounds. The expert authority coefficient for the consultation was 0.85 (Table 3 and table 4).

Table 3：Active participation of Delphi expert consultants

| Round | Number of questionnaires distributed | Returned questionnaires | Questionnaire response rate | Questionnaire modification suggestion rate |
| --- | --- | --- | --- | --- |
| 1 | 25 | 25 | 100% | 36% |
| 2 | 25 | 24 | 96% | 25% |

| Table 4：Summary of Delphi Expert Consultation Results | | | | | |
| --- | --- | --- | --- | --- | --- |
| Round | CV | Kendall's W | Expert judgment coefficient | Expert authority coefficient | Expert familiarity |
| 1 | ①＞0.25 | 0.149 | 1.000 | 0.944 | 0.888 |
| 2 | ②＞0.25 | 0.130 | 0.925 | 0.845 | 0.75 |
| note：① In the initial round of the Delphi expert consultation, the coefficient of variation for the statement "Insufficient water intake can cause OH" exceeded 0.25, so it was deleted. ② The findings from the second round of the Delphi expert consultation indicated that the coefficient of variation for the item "Taking hypoglycemic drugs can cause OH" was greater than 0.25, as was the coefficient of variation for the item "Taking anti-anxiety drugs can cause OH," leading to the exclusion of both items. | | | | | |

**Summary of Expert Consultation Feedback from the First Round:** A total of nine experts suggested revisions, which can be categorized into the following three main points: 1. The statement "Repeated use of antidepressants can cause OH" was amended to refer to "anti-anxiety drugs" instead. 2. The statement " If I take medication, I will concern about medication affecting my blood pressure and causing dizziness." was initially proposed for deletion. 3. The statement "I believe that OH is not harmful to the body" was removed. However, following further discussion and a review of the literature, it was decided that the statement " If I take medication, I will concern about medication affecting my blood pressure and causing dizziness." should be retained. This decision was primarily based on evidence indicating that many medications can indeed influence blood pressure and cause dizziness[1].

**Summary of expert consultation feedback from the second round:** 1. It was suggested that the statements "Taking antidepressants can cause OH" and "Taking anti-anxiety drugs can cause OH" be consolidated into a single sentence. 2. The recommendation was made to remove the sentence "Taking anti-anxiety drugs can cause orthostatic hypotension."

Consequently, the statements were merged into: "Taking antidepressants and anti-anxiety drugs can cause OH." The final questionnaire, consisting of 29 items addressing three domains—knowledge, attitudes, and practices—was subsequently evaluated for validity and reliability (Supplementary document 2).

**3. Logistic regression analysis results for orthostatic hypotension KAPQ scores**

The developed ordered logistic regression model met the proportional odds assumption (χ² = 44.89, p = 0.27), and no multicollinearity was detected among the independent variables included in the analysis (tolerance > 0.1, variance inflation factor < 10). Consequently, ordered logistic regression was employed in this study for factor analysis. The results indicated that individuals with a college education or higher had a lower risk of low knowledge, attitudes and practices scores than did those with a high school education or less (OR: 0.67, 95% CI: 0.55‒0.82). Similarly, those who received health education on OH had a lower risk of low scores in the Q25 range than those who did not (OR: 0.36, 95% CI: 0.30‒0.45). Additionally, a higher FES-I score was associated with a reduced risk of low knowledge and practices scores in the Q25 range (OR: 0.98, 95% CI: 0.97‒0.99) (table 5).

Table 5: Logistic regression analysis results for orthostatic hypotension KAPQ scores

| Items | B | Standard errors | Exp(B) | 95%CI | | p |
| --- | --- | --- | --- | --- | --- | --- |
|  |  |  |  | Lower limit | Upper limit |  |
| Education background |  |  |  |  |  |  |
| High school & below | 0.402 | 0.101 | 1.494 | 1.226 | 1.821 | <0.001 |
| College degree & higher | 0 |  | 1.0 |  |  |  |
| Health education related to OH |  |  |  |  |  |  |
| Yes | -1.013 | 0.104 | 0.363 | 0.296 | 0.445 | <0.001 |
| No | 0 |  | 1.0 |  |  |  |
| FES-I score | -0.019 | 0.004 | 0.981 | 0.973 | 0.989 | <0.001 |
|  |  |  |  |  |  |  |

**References**

1. Association, G.P., *Expert Consensus on the Prevention and Management of Drug-Related Falls in Older Adults.* Pharmacy Today, 2019. **29**(10): p. 649-658.
