# Supplementary material for: Validation of the orthostatic hypotension knowledge, attitudes, and practices questionnaire and investigation of influencing factors: a cross-sectional study
Source: Front Public Health. 2025 Oct 14;13:1561758. doi: 10.3389/fpubh.2025.1561758 (PMC12560239; doi:10.3389/fpubh.2025.1561758)
Supplement: Supplementary file 2 [file Table_2.DOCX]

The orthostatic hypotension knowledge, attitudes, practices questionnaire (KAPQ)

1. Have you received health education related to orthostatic hypotension (referred to as receiving professional education from medical personnel)?

○ Yes

○ No

1. Knowledge dimension

|  | Strongly disagree | Disagree | Somewhat agree | Agree | Strongly agree |
| --- | --- | --- | --- | --- | --- |
| （1）Taking antidepressants and anti-anxiety drugs can cause OH. | 1 | 2 | 3 | 4 | 5 |
| （2）Taking sedative and hypnotic drugs can cause OH. | 1 | 2 | 3 | 4 | 5 |
| （3）Taking diuretics can cause OH. | 1 | 2 | 3 | 4 | 5 |
| （4）Sudden positional changes (sitting up or getting up) can lead to OH. | 1 | 2 | 3 | 4 | 5 |
| （5）OH can lead to falls. | 1 | 2 | 3 | 4 | 5 |
| （6）Certain chronic conditions such as cardiovascular disease or diabetes increase the risk of OH. | 1 | 2 | 3 | 4 | 5 |
| （7）Post-bath or heavy sweating activities increase the risk of OH. | 1 | 2 | 3 | 4 | 5 |
| （8）Weakness and fatigue increase the risk of OH. | 1 | 2 | 3 | 4 | 5 |
| （9）OH may occur when you feel dizzy after changing positions or standing for a long time. | 1 | 2 | 3 | 4 | 5 |
| （10）OH may occur when you experience dizziness, blurred vision, or a black haze upon changing positions or standing for a long time. | 1 | 2 | 3 | 4 | 5 |
| （11）OH may occur when you experience weakness of the lower extremities after changing positions or standing for a long time. | 1 | 2 | 3 | 4 | 5 |
| （12）OH may occur when you experience nausea or vomiting after changing positions or standing for a long time. | 1 | 2 | 3 | 4 | 5 |
| （13）OH may occur when you experience dyspnea after changing positions or standing for a long time. | 1 | 2 | 3 | 4 | 5 |

1. Attitudes Dimension

|  | Strongly disagree | Disagree | Somewhat agree | Agree | Strongly agree |
| --- | --- | --- | --- | --- | --- |
| (1) I believe that OH can cause falls. | 1 | 2 | 3 | 4 | 5 |
| (2) I believe that implementing management measures can lower the risk of OH. | 1 | 2 | 3 | 4 | 5 |
| (3) I believe that OH management is important. | 1 | 2 | 3 | 4 | 5 |
| (4) I'd like to learn about the risk factors of OH. | 1 | 2 | 3 | 4 | 5 |
| (5) I'd like to learn about the symptoms of OH. | 1 | 2 | 3 | 4 | 5 |
| 1. I'd like to learn about the preventive measures for OH. | 1 | 2 | 3 | 4 | 5 |
| (7) I'm worried about getting OH. | 1 | 2 | 3 | 4 | 5 |

1. Practices Dimension

|  | Strongly disagree | Disagree | Somewhat agree | Agree | Strongly agree |
| --- | --- | --- | --- | --- | --- |
| (1) I will proactively take measures to prevent OH and lower risk of falls. | 1 | 2 | 3 | 4 | 5 |
| (2) I will stand up slowly to give my body an adjustment to prevent OH. | 1 | 2 | 3 | 4 | 5 |
| (3) I will drink plenty of water, especially after excessive sweating to prevent OH. | 1 | 2 | 3 | 4 | 5 |
| (4) I will wear lower limb compression socks to prevent OH. | 1 | 2 | 3 | 4 | 5 |
| (5) I will avoid drinking alcohol. | 1 | 2 | 3 | 4 | 5 |
| (6) I will do daily exercise. | 1 | 2 | 3 | 4 | 5 |
| (7) I will follow my health care provider's advice to prevent OH. | 1 | 2 | 3 | 4 | 5 |
| (8) I will learn how to prevent OH. | 1 | 2 | 3 | 4 | 5 |
| (9) If I take medication, I will concern about medication affecting my blood pressure and causing dizziness. | 1 | 2 | 3 | 4 | 5 |
