# Supplementary material for: Validation of the orthostatic hypotension knowledge, attitudes, and practices questionnaire and investigation of influencing factors: a cross-sectional study
Source: Front Public Health. 2025 Oct 14;13:1561758. doi: 10.3389/fpubh.2025.1561758 (PMC12560239; doi:10.3389/fpubh.2025.1561758)
Supplement: Supplementary file 3 [file Table_3.DOCX]

**Supplementary document 3:** **Results of factor analysis and regression analysis**

***The results of exploratory factor analysis (EFA)***

**Presentation of project analysis results**

（1）critical ratio method

The critical ratio method involves calculating the critical ratio (CR) for each item in the questionnaire and subsequently removing or modifying items whose CR values do not achieve statistical significance. In this study, the top 27% and bottom 27% of participants were classified as the high-score and low-score groups, respectively. An independent samples t-test was conducted to compare the mean scores between these groups for each item. Items that did not exhibit statistically significant t-test results were excluded. The findings indicated that all items demonstrated discriminative power, evidenced by significant score differences between the high- and low-score groups. Consequently, all 29 items were retained (Table 1).

Table 1: Analysis results of the critical ratio of high and low KAPQ scores

| Items | CR | p |  | Items | CR | p |
| --- | --- | --- | --- | --- | --- | --- |
| 1 | -28.329 | 0.000 |  | 16 | -39.037 | 0.000 |
| 2 | -30.564 | 0.000 |  | 17 | -35.785 | 0.000 |
| 3 | -30.380 | 0.000 |  | 18 | -34.563 | 0.000 |
| 4 | -34.929 | 0.000 |  | 19 | -34.125 | 0.000 |
| 5 | -36.979 | 0.000 |  | 20 | -28.510 | 0.000 |
| 6 | -39.371 | 0.000 |  | 21 | -34.580 | 0.000 |
| 7 | -39.935 | 0.000 |  | 22 | -33.270 | 0.000 |
| 8 | -39.532 | 0.000 |  | 23 | -33.046 | 0.000 |
| 9 | -38.963 | 0.000 |  | 24 | -24.407 | 0.000 |
| 10 | -38.681 | 0.000 |  | 25 | -21.807 | 0.000 |
| 11 | -39.043 | 0.000 |  | 26 | -24.360 | 0.000 |
| 12 | -39.041 | 0.000 |  | 27 | -27.419 | 0.000 |
| 13 | -37.706 | 0.000 |  | 28 | -28.572 | 0.000 |
| 14 | -32.830 | 0.000 |  | 29 | -29.574 | 0.000 |
| 15 | -34.276 | 0.000 |  |  |  |  |

(2) factor analysis

Conduct homogeneity tests to evaluate the items, calculating the correlation coefficient between each item and the total score. A higher correlation coefficient signifies a stronger association between the item and the total score, indicating better homogeneity. Conversely, a lower correlation coefficient suggests poorer homogeneity, warranting consideration for item removal. The Spearman correlation analysis results in this study demonstrate that all correlation coefficients between each item and the total score of the KAPQ questionnaire exceed 0.5. This finding indicates that the items exhibit high homogeneity with the overall questionnaire, and thus, no items require deletion. For further details (Table 2).

Table 2: Correlation analysis between KAPQ items and total scores

| Items | r | p |  | Items | r | p |
| --- | --- | --- | --- | --- | --- | --- |
| 1 | 0.700 | <0.01 |  | 16 | 0.804 | <0.01 |
| 2 | 0.723 | <0.01 |  | 17 | 0.772 | <0.01 |
| 3 | 0.716 | <0.01 |  | 18 | 0.757 | <0.01 |
| 4 | 0.762 | <0.01 |  | 19 | 0.752 | <0.01 |
| 5 | 0.782 | <0.01 |  | 20 | 0.663 | <0.01 |
| 6 | 0.818 | <0.01 |  | 21 | 0.759 | <0.01 |
| 7 | 0.819 | <0.01 |  | 22 | 0.732 | <0.01 |
| 8 | 0.816 | <0.01 |  | 23 | 0.729 | <0.01 |
| 9 | 0.807 | <0.01 |  | 24 | 0.591 | <0.01 |
| 10 | 0.818 | <0.01 |  | 25 | 0.549 | <0.01 |
| 11 | 0.811 | <0.01 |  | 26 | 0.577 | <0.01 |
| 12 | 0.810 | <0.01 |  | 27 | 0.631 | <0.01 |
| 13 | 0.801 | <0.01 |  | 28 | 0.657 | <0.01 |
| 14 | 0.762 | <0.01 |  | 29 | 0.683 | <0.01 |
| 15 | 0.781 | <0.01 |  |  |  |  |

Spearman's correlation analysis was employed to examine the relationship between each item of the KAPQ and its respective dimension. The findings revealed that the correlation coefficients for items within the knowledge dimension and the overall knowledge dimension score exceeded 0.8. Similarly, the correlation coefficients for items within the attitude dimension and the overall attitude dimension score surpassed 0.7, as did those within the behavior dimension and its total score, indicating strong correlations (Table 3).

Table 3: Correlation analysis between KAPQ items and corresponding dimension total scores

| Items | Knowledge dimension score(r) | p |  | Items | Attitudes dimension score (r) | p |  | Items | Practices dimension score (r) | p |
| --- | --- | --- | --- | --- | --- | --- | --- | --- | --- | --- |
| 1 | 0.802 | <0.01 |  | 14 | 0.837 | <0.01 |  | 21 | 0.843 | <0.01 |
| 2 | 0.828 | <0.01 |  | 15 | 0.866 | <0.01 |  | 22 | 0.818 | <0.01 |
| 3 | 0.814 | <0.01 |  | 16 | 0.895 | <0.01 |  | 23 | 0.847 | <0.01 |
| 4 | 0.833 | <0.01 |  | 17 | 0.888 | <0.01 |  | 24 | 0.729 | <0.01 |
| 5 | 0.813 | <0.01 |  | 18 | 0.888 | <0.01 |  | 25 | 0.728 | <0.01 |
| 6 | 0.855 | <0.01 |  | 19 | 0.884 | <0.01 |  | 26 | 0.808 | <0.01 |
| 7 | 0.872 | <0.01 |  | 20 | 0.786 | <0.01 |  | 27 | 0.807 | <0.01 |
| 8 | 0.859 | <0.01 |  |  |  |  |  | 28 | 0.865 | <0.01 |
| 9 | 0.855 | <0.01 |  |  |  |  |  | 29 | 0.845 | <0.01 |
| 10 | 0.860 | <0.01 |  |  |  |  |  |  |  |  |
| 11 | 0.871 | <0.01 |  |  |  |  |  |  |  |  |
| 12 | 0.861 | <0.01 |  |  |  |  |  |  |  |  |
| 13 | 0.850 | <0.01 |  |  |  |  |  |  |  |  |

***The results of confirmatory factor analysis (CFA)***

This dataset comprises three factors, 29 variables, and 1,488 samples, satisfying the fundamental data prerequisites for conducting confirmatory factor analysis (table 4).

**Table 4: Summary of basic factors**

| Factors | n |
| --- | --- |
| Knowledge dimension | 13 |
| Attitudes Dimension | 7 |
| Practices Dimension | 9 |
| Total items | 29 |
| Sample size | 1488 |

As evidenced by the factor loading coefficients presented in table 5, the standardized loading coefficients for each measurement item within the knowledge, attitude, and behavior dimensions all exceed 0.6. This indicates that they possess adequate variance explanation rates, thereby demonstrating that each variable can be represented on the same factor.

**Table 5:** **the factor loading coefficient table**

| Factors | items | Non-standard load factor | Standard load factor | z | S.E. | P |  |
| --- | --- | --- | --- | --- | --- | --- | --- |
| Knowledge dimension | Item1‒13 | 1 1.051 1.02 1.094 1.104 1.134 1.162 1.156 1.148 1.162 1.175 1.165 1.152 | 0.727 0.768 0.753 0.814 0.838 0.873 0.889 0.895 0.901 0.904 0.906 0.872 0.855 | - 30.2 29.567 32.151 33.149 34.66 35.354 35.612 35.85 36.006 36.075 34.62 33.873 | - 0.035 0.035 0.034 0.033 0.033 0.033 0.032 0.032 0.032 0.033 0.034 0.034 | - 0.000*** 0.000*** 0.000*** 0.000*** 0.000*** 0.000*** 0.000*** 0.000*** 0.000*** 0.000*** 0.000*** 0.000*** |  |
| Attitudes Dimension | Item14‒20 | 1 1.049 1.087 1.108 1.094 1.09 0.897 | 0.794 0.837 0.88 0.905 0.909 0.904 0.702 | - 37.488 40.205 41.934 42.202 41.867 29.715 | - 0.028 0.027 0.026 0.026 0.026 0.03 | - 0.000*** 0.000*** 0.000*** 0.000*** 0.000*** 0.000*** |  |
| Practices Dimension | Item21‒29 | 1 0.899 0.993 0.918 0.687 0.84 0.763 1.028 0.914 | 0.877 0.856 0.85 0.649 0.642 0.759 0.776 0.842 0.818 | - 45.566 44.948 28.94 28.49 36.724 38.062 44.18 41.791 | - 0.02 0.022 0.032 0.024 0.023 0.02 0.023 0.022 | - 0.000*** 0.000*** 0.000*** 0.000*** 0.000*** 0.000*** 0.000*** 0.000*** |  |
| Note: ***, **, and * represent significance levels of 1%, 5%, and 10%, respectively. | | | | | | |  |

The findings from the model's AVE and CR tests, as presented in table 6, indicate the following: For the knowledge dimension, the average variance extracted (AVE) is 0.715, and the composite reliability (CR) is 0.97. For the attitude dimension, the AVE is 0.719, and the CR is 0.947. For the behavior dimension, the AVE is 0.615, and the CR is 0.934. These results demonstrate that the measurement indicators within each factor exhibit excellent levels of extraction.

**Table 6: Model evaluation**

| Factor | AVE | CR |
| --- | --- | --- |
| Knowledge dimension | 0.715 | 0.97 |
| Attitudes Dimension | 0.719 | 0.947 |
| Practices Dimension | 0.615 | 0.934 |

Table 7 presents the findings of the Pearson correlation analysis conducted between the factors and the square root of the Average Variance Extracted (AVE). The results indicate that each dimension demonstrates relatively strong discriminant validity.

**Table 7: Pearson correlation and AVE square root values**

|  | Knowledge dimension | Attitudes Dimension | Practices Dimension |
| --- | --- | --- | --- |
| Knowledge dimension | 0.846 |  |  |
| Attitudes Dimension | 0.712(0.000***) | 0.848 |  |
| Practices Dimension | 0.579(0.000***) | 0.766(0.000***) | 0.784 |
| Note: ***, **, and * represent significance levels of 1%, 5%, and 10%, respectively. The values located on the diagonal represent the square root of the AVE for each factor. | | | |

A Goodness of Fit Index (GFI) of 0.9 or higher suggests an adequate model fit. A Root Mean Square Error of Approximation (RMSEA) value below 0.1 is considered acceptable, with lower values indicating a better fit. A Root Mean Square Residual (RMR) of less than 0.1 also signifies a satisfactory model fit. A Comparative Fit Index (CFI) of 0.9 or above is indicative of a good model fit. For both the Non-Normed Fit Index (NNFI) and the CFI, higher values correspond to a better-fitting model, as detailed in table 8.

**Table 8: Model fitting indicators**

| Commonly used indicators | X² | df | P | Chi-square degrees of freedom ratio | GFI | RMSEA | RMR | CFI | NFI | NNFI |
| --- | --- | --- | --- | --- | --- | --- | --- | --- | --- | --- |
| judgment criteria | - | - | >0.05 | <3 | >0.9 | <0.10 | <0.05 | >0.9 | >0.9 | >0.9 |
| value | 8607.695 | 374 | 0 | 23.015 | 0.829 | 0.122 | 0.055 | 0.835 | 0.829 | 0.821 |
| Note: ***, **, and * represent significance levels of 1%, 5%, and 10%, respectively. | | | | | | | | | | |

The findings from the covariance analysis, as presented in table 9, indicate that the standardized estimated coefficient between the knowledge and attitude dimensions is 0.731, between the knowledge and behavior dimensions is 0.624, and between the attitude and behavior dimensions is 0.834. These coefficients collectively demonstrate a strong correlation among the dimensions.

**Table 9: Factor covariance table**

| Factor A | Factor B | non-standard estimation coefficient | standard error | z | P | standard estimated coefficient |
| --- | --- | --- | --- | --- | --- | --- |
| Knowledge dimension | Attitudes Dimension | 0.381 | 0.021 | 18.265 | 0.000*** | 0.731 |
| Knowledge dimension | Practices Dimension | 0.369 | 0.021 | 17.36 | 0.000*** | 0.624 |
| Attitudes Dimension | Practices Dimension | 0.47 | 0.023 | 20.868 | 0.000*** | 0.834 |
| Note: ***, **, and * represent significance levels of 1%, 5%, and 10%, respectively. | | | | | | |

The findings from the confirmatory factor analysis indicated that the dimensions of knowledge, attitude, and behavior, along with each individual item, demonstrated robust discriminant validity.

***Logistic regression analysis results for orthostatic hypotension KAPQ scores***

The developed ordered logistic regression model met the proportional odds assumption (χ² = 44.89, p = 0.27), and no multicollinearity was detected among the independent variables included in the analysis (tolerance > 0.1, variance inflation factor < 10). Consequently, ordered logistic regression was employed in this study for factor analysis. The results indicated that individuals with a college education or higher had a lower risk of low knowledge, attitudes and practices scores than did those with a high school education or less (OR: 0.67, 95% CI: 0.55‒0.82). Similarly, those who received health education on OH had a lower risk of low scores in the Q25 range than those who did not (OR: 0.36, 95% CI: 0.30‒0.45). Additionally, a higher FES-I score was associated with a reduced risk of low knowledge and practices scores in the Q25 range (OR: 0.98, 95% CI: 0.97‒0.99) (table10).

Table 10: Logistic regression analysis results for orthostatic hypotension KAPQ scores

| Items | B | Standard errors | Exp(B) | 95%CI | | p |
| --- | --- | --- | --- | --- | --- | --- |
|  |  |  |  | Lower limit | Upper limit |  |
| Education background |  |  |  |  |  |  |
| High school & below | 0.402 | 0.101 | 1.494 | 1.226 | 1.821 | <0.001 |
| College degree & higher | 0 |  | 1.0 |  |  |  |
| Health education related to OH |  |  |  |  |  |  |
| Yes | -1.013 | 0.104 | 0.363 | 0.296 | 0.445 | <0.001 |
| No | 0 |  | 1.0 |  |  |  |
| FES-I score | -0.019 | 0.004 | 0.981 | 0.973 | 0.989 | <0.001 |
|  |  |  |  |  |  |  |

**References**
[1] Scientific Platform Serving for Statistics Professional 2021. SPSSPRO. (Version 1.0.11)[Online Application Software]. Retrieved from https://www.spsspro.com.
